# Stereoscopic projection lithography based 3D printing with high precision for advanced tissue engineering application

**DOI:** 10.3389/fbioe.2022.1074157

**Published:** 2022-11-18

**Authors:** Jianli Ma, Shuo Zhao, Yongcheng Li, Jingjing Hu, Ling Zhang, Xuan Zhou, Li Yan

**Affiliations:** ^1^ Shenzhen Sunshine Laser and Electronics Technology Co. Ltd, Shenzhen, China; ^2^ Department of Geriatric Medicine, The Second Medical Center and National Clinical Research Center for Geriatric Diseases, Chinese PLA General Hospital, Beijing, China; ^3^ Key Lab of Semiconductor Materials Science, Institute of Semiconductors, Chinese Academy of Sciences, Beijing, China; ^4^ Shenzhen 3D Printing Manufacturing Innovation Center, Shenzhen, China

**Keywords:** stereoscopic projection lithography, 3D printing, GelMA, micro structure scaffold, adipose tissue-derived stromal cells (ADSC)

## Abstract

The emergence of tissue engineering technology provides an option for the treatment of early organ and tissue lesions by combination of biomimetic scaffolds and stem cells. Stereoscopic projection lithography is utilized broadly in varied application areas due to its high-precision, resolution, and efficiency features. It can be used to fabricate and manufacture complex scaffolds with hierarchical construct, which are highly suitable for advanced tissue engineering application. In current work, gelatin methacrylate (GelMA) was synthesized and fabricated to bioactive scaffold because of its excellent biocompatibility and biodegradability by using stereoscopic projection lithography based 3D printer (YC-M3D-10). The scaffold displayed multilayered micro structures that supported stem cell growth and promoted cell proliferation. The results demonstrated that the cells proliferated significantly on the printed GelMA scaffold after 6 days. Moreover, GelMA scaffolds can promote cell proliferation and show great prospects in future tissue engineering applications.

## Introduction

With the rapid development of modern medicine, organ failure is still an intractable issue in clinical treatment. Some tissues, such as liver, are able to restrictedly regenerate due to its unique features. However, organ transplantation is considered as the only effective strategy in clinic. Nevertheless, the transplantable allografts are restricted by insufficient donor sources and potential immune rejection. Therefore, organ/tissue regeneration and repairing are still a significant clinical challenge.

Compared with traditional methods, the emergence of tissue engineering technology provides a promising option for treatment of organ/tissue injury by combination of complex biomimetic scaffolds and stem cells. Scaffolds (such as electrostatic spun nanofibers, hydrogels and 3D catheters), including biocompatible natural (silk, chitosan, gelatin, *etc.*) and synthetic biomaterials (polylactic acid, poly (lactic acid glycolic acid), and polyvinyl alcohol, *etc.*), have been widely used in tissue engineering for decades. Universally, cells were cultured in plate and then were induced into differentiation in traditional tissue engineering applications. Recently, many researches pay close attentions in the limitation of the classical cell culture procedures that there have some differences in the behavior and differentiation of cell between plate-cultured and *in situ* cell in body. The phenomena were considered to relate with three-dimensional environment of *in situ* cell of body. Cell polarity plays an crucial role in cell proliferation and differentiation, which involves to the asymmetric distribution of organelles and functions in the cell. Moreover, cell polarity determines the fate and behavior of cells (Florian and Geiger, 2010). Therefore, three-dimensional environment takes an essential part in cell development, and the 3D printed scaffold structure is a key role for tissue/organ regeneration (Butler and Wallingford, 2017).

However, traditional tissue engineering techniques are still not satisfactory in clinical application due to some limitations, such as low precision, many manufacturing processes, high cost and low efficiency (Anada et al., 2019). More importantly, it is intricate to customize the optimal alternative structure to fit the different clinical tissue defects. Recently, the advanced 3D biological printing technologies attract more and more focuses on tissue engineering application, which has great potential in the manufacture of complex and customizable 3D biomedical quasi-organs. In the field of biomedicine application, 3D printing technology provides many merits in using computer-aided design (CAD) to produce high-precision, low-cost and innovative scaffolds with complex 3D microstructure. Digital Light Processing (DLP) is a 3D biological printing technology with high precision and resolution, which utilizes a rapid prototyping lithography technology to polymerize or cross-link photocurable polymer ink in layer by layer method under the specific light beam exposure (Lim et al., 2018; Zhang et al., 2021). Particularly, DLP based technologies were employed to fabricate biomimetic tissues, including bone, blood vessels, nerve scaffold, and other tissues for tissue repair applications (Chen et al., 2021; Jiang et al., 2021; Schoonraad et al., 2021; Wang et al., 2021; Chen et al., 2022; Martinez et al., 2022). In the current work, high-precision DLP printers have been used to create a unique tissue engineering matrix (Martinez et al., 2022; Xie et al., 2022).

Biocompatibility is an essential prerequisite prior to biomedicine application. GelMA is a gelatin derivative that contains tremendous arginine-glycine-aspartic acid (RGD) and matrix metalloproteinases sequences (Lewis et al., 2018). GelMA has excellent biocompatibility and biodegradability and has been utilized to prepare 3D printed tissue scaffolds for effectively promoting cell growth (Cuvellier et al., 2021; Elkhoury et al., 2021; Su et al., 2021). The purpose of current study is to fabricate a 3D GelMA scaffolds for tissue engineering application using polarization modulated stereo digital lithography technology method. Currently, the 3D printing GelMA scaffold with hierarchical structure is manufactured using our custom designed DLP printer. Thus, stem cells were cultured on scaffolds and proliferation behavior was observed.

## Materials and methods

### Synthesis of GelMA

GelMA was synthesized as described in our previous work with minor modification (Zhou et al., 2016; Zhou et al., 2017; Zhou et al., 2018; Zhou et al., 2019; Zhou et al., 2020a; Zhou et al., 2020b; Herreros-Pomares et al., 2021). Briefly, 10 g of gelatin (Type A, Sigma-Aldrich, United States) was fully dissolved into 100 ml pure water with constant magnetic stirring at 60°C for about 30 min. Next, 4 ml methacrylic anhydride (Sigma-Aldrich, United States) was added drop-wise into the gelatin solution with constant magnetic stirring at 50°C for another 3 h. After that, the mixture was then dialyzed with dialysis bags (8–14 kDa cut-off) against pure water to remove impurities at 40°C for 5 days. Finally, GelMA was obtained through lyophilization.

In order to prepare GelMA ink, 8 g of GelMA was dissolved in 100 ml pure water containing 0.5% (w/v) lithium phenyl (2,4,6-trimethylbenzoyl)phosphinate (LAP) (Sigma-Aldrich) which was used as a photoinitiator. Finally, the GelMA ink was prepared and stored at 4°C.

### 3D printer

The 3D printer, model YC-M3D-10 (manufactured by Shenzhen sunshine Laser & Electronics Technology Co., Ltd.), was employed to fabricate GelMA scaffold. Based on micron high-precision digital lithography technology, YC-M3D-10 consists of 4 K resolution (4,000 × 2,400 pixel) polarization modulator, optical and electronical control auxiliary system and software systems. It can improve the overall printing accuracy and lamination up to 7 μm of optical resolution. Compared with the traditional DLP printer, the YC-M3D-10 printing scaffold has higher resolution and printing precision, the micro structure of scaffold can be generated. The resultant scaffold is more conducive to the growth of stem cells.

### 3D printed GelMA scaffolds

In order to obtain optimal scaffold, The parameters of the 3D printer were set as follow. Exposure wavelength: 405 nm, optical power density: 17mw/cm^2^, thickness of each exposure layer: 40 μm, exposure time of each layer: 0.7s. The bio ink was placed in the printer reservoir, and scaffold model was printed in layer by layer using laser exposure. After that, the printed scaffold was rinsed in 100°C water followed by freeze-dried, and it was soaked in 100°C distilled water for 20 min prior to use in experiment.

### Cell culture

The adipose mesenchymal stem cells (ADSCs) were extracted from BALB/c suckling mice (SPF) (Beijing, BIOTECHNOLOGY Co., Ltd.). The ADSC were cultured in MEM-F12 medium supplemented with 10% (v/v) fetal bovine serum for cell passage cultivation.

Prior to cell seeding, 3D scaffolds were placed in 24-well plates and disinfected with cobalt 60. It was soaked in 100°C distilled water for 20min prior to use in experiment. The cells were seeded directly on the GelMA scaffolds at a density of 1 × 10^6^ cells/well and continuously cultured for 6 days. At predetermined time intervals, the scaffolds were harvested for subsequent experiments. The proliferation and viability of cell on 3D GelMA scaffold were investigated for 6 days. All cells were incubated in a 95% humidified atmosphere, with 5% CO_2_, at 37°C.

### Sample preparation

Sample preparation for scanning electron microscope (SEM): the samples were taken and washed with PBS for three times, and then fixed with 2.5% glutaraldehyde solution for 4 h. After that, the samples were dehydrated by ethanol with serial different concentration (50%, 60%, 70%, 80%, 90%, 100%), each step for 10 min and repeated twice. Next, the samples were immersed in isoamyl acetate for 40 min, in hexamethyl disilylamine for 8–10 min, and dried by critical point method, followed by gold spray coating before SEM observation.

Sample preparation for Confocal with CMFDA-PI fluorescence staining: After culturing 6 days, the samples were taken out and washed 3 times with PBS, stained with CMFDA dye solution for 5 min, washed with PBS, and soaked in PI dye solution for another 5 min. Finally, the samples were observed by confocal microscope (A1/LSM-Kit).

### Characterization of cell cultured on scaffold

The morphology of GelMA scaffold was observed by SEM for 6 days. Cell survival on the scaffolds was observed by confocal microscopy at the first and third days. GelMA scaffold samples were harvested and stained with live/dead cell viability detection reagent (CMFDA-PI staining). Under confocal fluorescence microscopy observation, the green and red fluorescence was considered as living cells dead cells, respectively. Each sample randomly selected four different regions in the field for average quantitative analysis. The color of the image was analyzed with ImageJ software. Cell viability was calculated by the following formula: total number of living cells/(total number of living cells + total number of dead cells) × 100%. At the same time, the panoramic jigsaw puzzle of confocal fluorescence microscope (vertical interval 200 μm) was used to scan the multi-layer fitting image. The whole cell distribution and cell density of the sample were observed.

## Results

### Synthesis and characterization of GelMA

**FIGURE 1:**
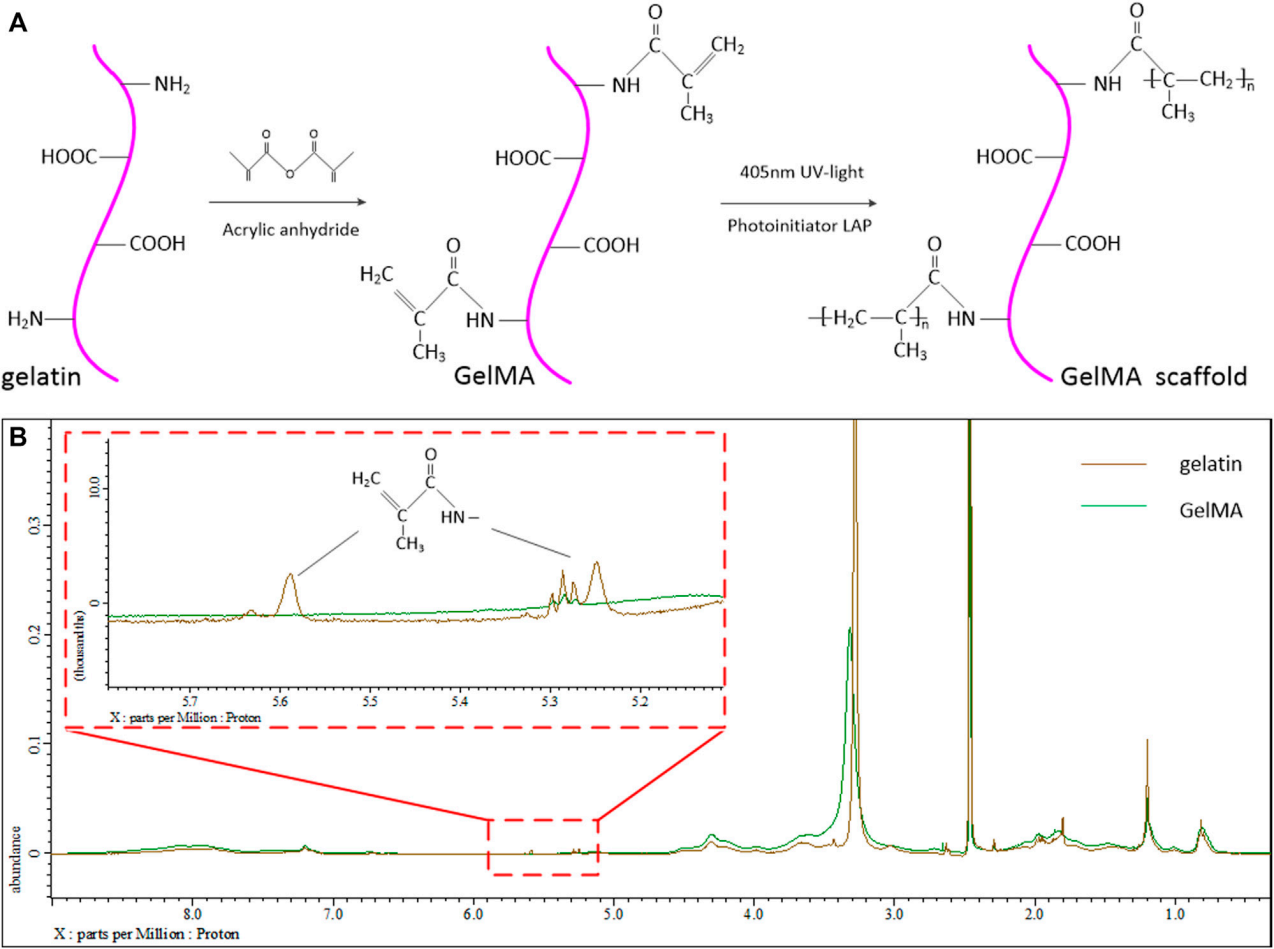
**(A)** Schematic diagram of the modification process and UV-curing process of GelMA. **(B)** 1H NMR spectrum of the gelatin and modified GelMA group.

Figure 1A shows schematic diagram of the modification process and UV-curing process of GelMA. The 1H NMR spectra of gelatin and GelMA are shown in Figure 1B. The methacrylate substituents were successfully introduced into the gelatin molecules. The new absorption peaks of 5.3 and 5.6 ppm were not obvious in GelMA 1H NMR spectra. The results can be explained that a few of amino groups in gelatin participated into the methacrylated reaction process. These new peaks are produced by the methacrylate groups modified by amide reaction in the process of gelatin synthesis. The 3.2 ppm is the solvent peak of deuterium DMSO. Although there have a few amount of C=C double bonds in GelMA, these double bonds can still be photopolymerized and crosslinked to hydrogel.

### 3D printed GelMA scaffold prointing process and characterization

**FIGURE 2:**
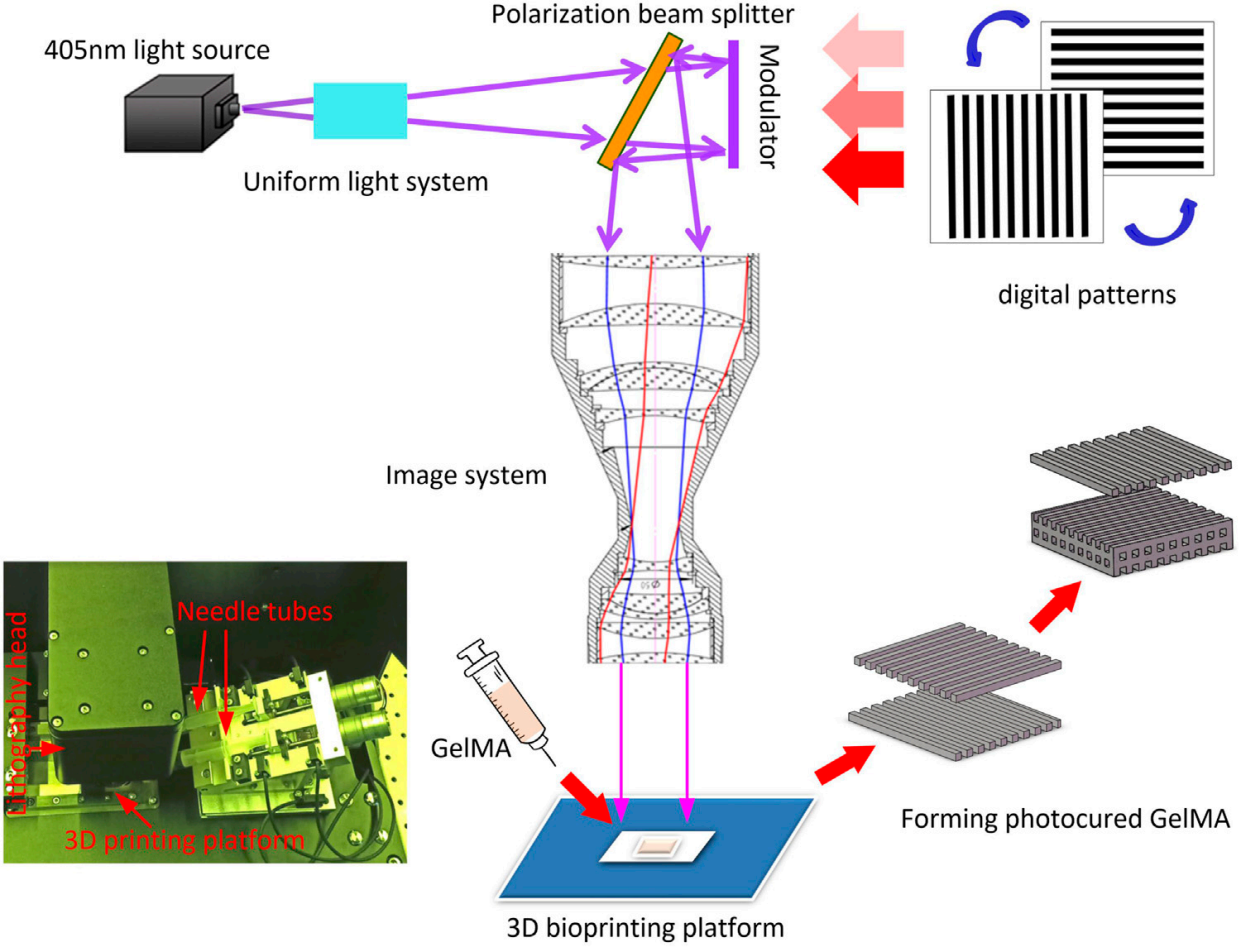
Schematic of 3D printer and printing process.

**FIGURE 3:**
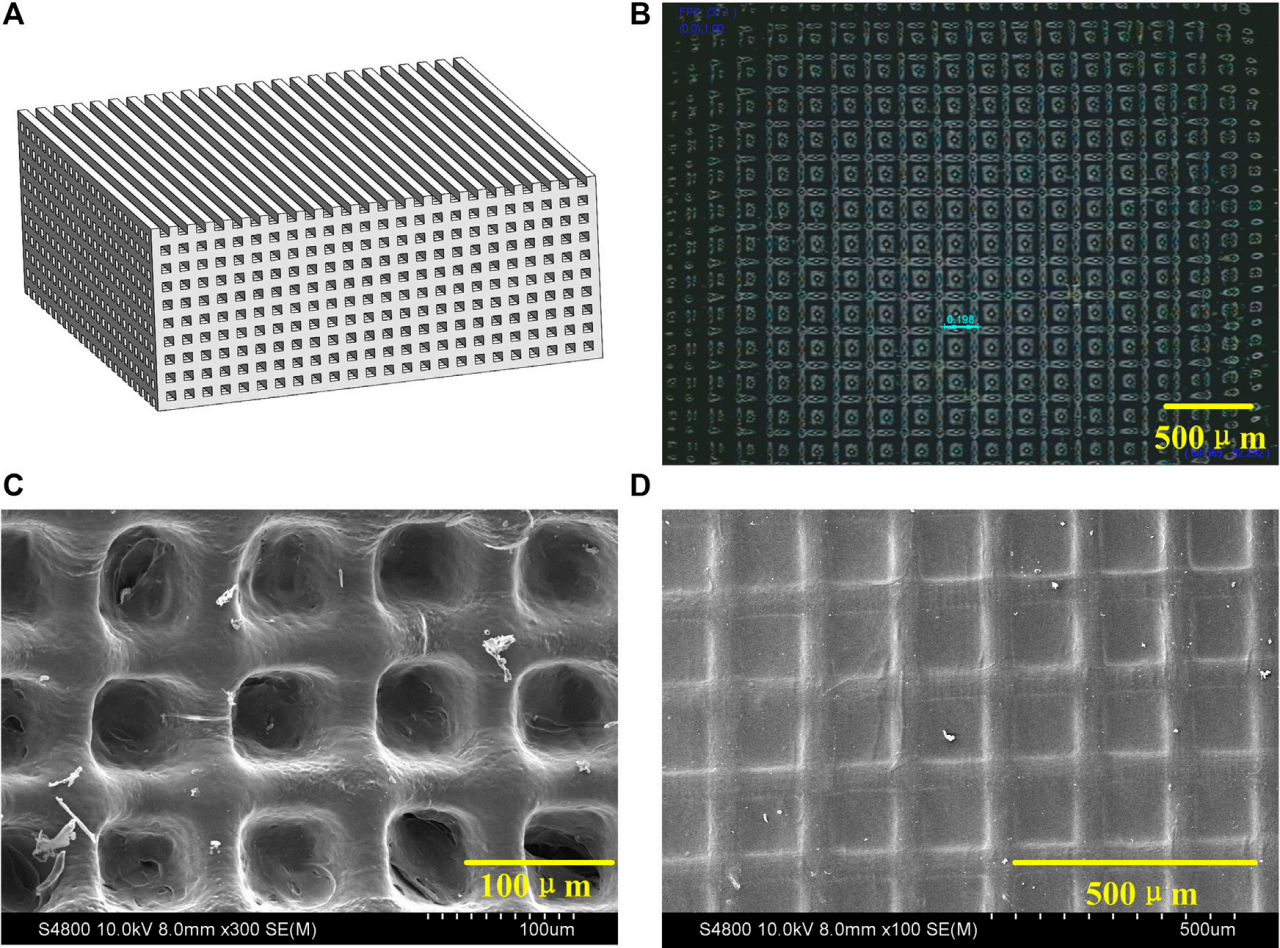
**(A)** CAD module of 3D scaffold. **(B)** Microscope image of 3D scaffold. **(C)** SEM micrograph of lyophilized scaffold. **(D)** SEM micrograph of air-dried scaffold.

The 3D printing process is shown in Figure 2. The 405 nm UV-light is irradiated on the polarization modulator through the uniform light system. The UV laser was modulated by polarization modulator into a digital patterns and projected onto the GelMA ink by the imaging system. The pre-designed CAD wood-pile structures (Figure 3A) were printed by alternately changing the digital patterns of the modulator The scaffolds with homogeneous pores (198 μm) and uniform channels were observed in Figure 3B, and quite matched with CAD pattern. Figures 3C,D shows the SEM morphologies of lyophilized and air-dried scaffold respectively. The porous structure in lyophilized scaffold is significant than that in air-dried scaffold. The structure difference allows cells and nutrients easier to penetrate scaffold that are able to promote cell proliferation or differentiation.

### Cell proliferation on 3D printed GelMA scaffold

**FIGURE 4:**
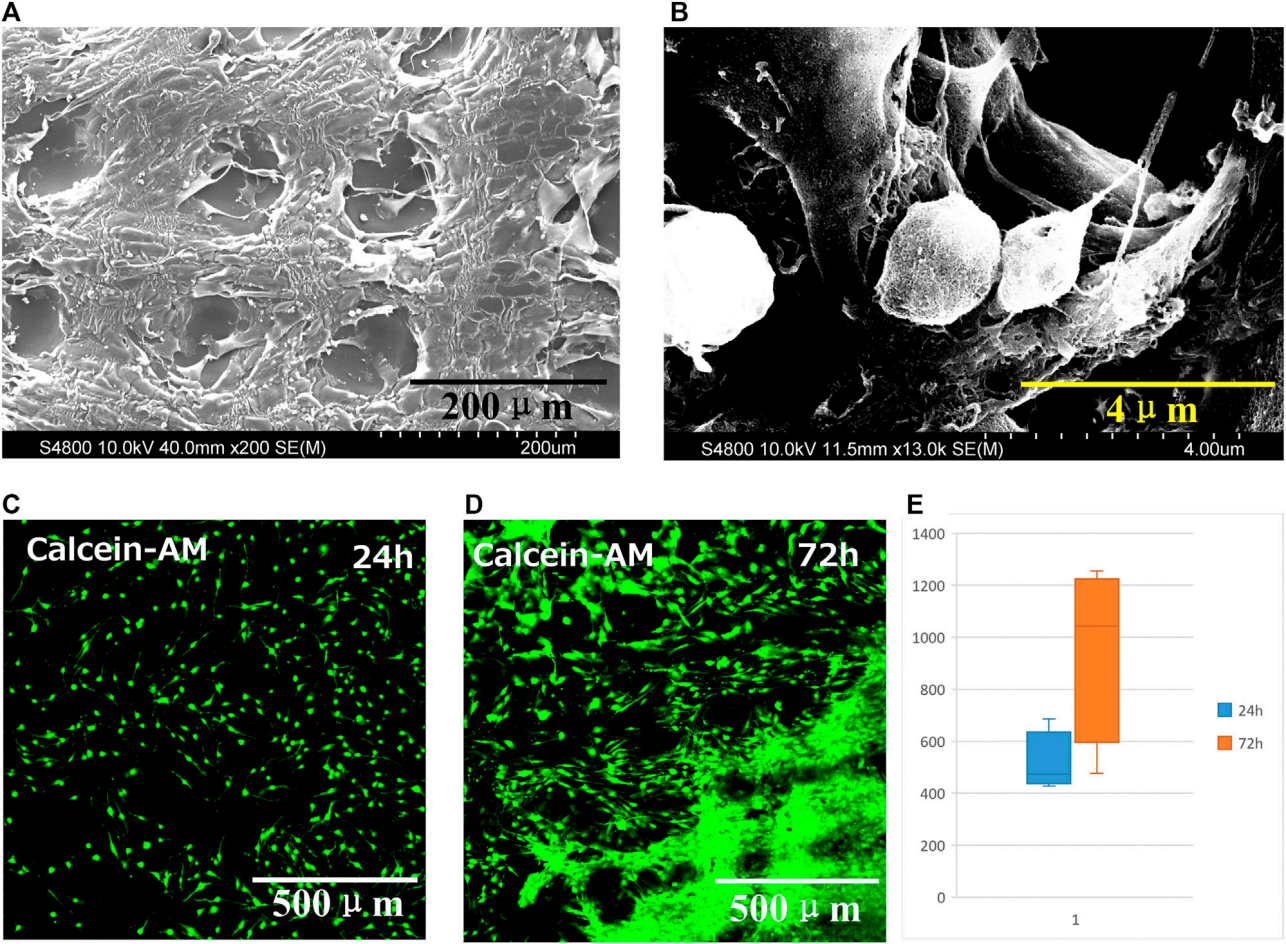
**(A)** SEM micrograph and **(B)** magnified SEM image of GelMA scaffold seeded with cells for 6 days. Fluorescence micrographs of dead and live cell staining on scaffold at **(C)** 24 h and **(D)** 72 h. **(E)** Cell proliferation on scaffold at 24 h and 72 h, data are mean ± standard deviation, *n* = 9.

The biocompatibility of 3D printed GelMA scaffold *in vitro* was investigated by culturing cells onto scaffolds. The interlaced network structure was shown in Figures 4A,B. The cell spread and stretched on scaffold with pseudopodia.

Fluorescence micrographs of dead and live cell staining on scaffold shown in Figures 4C,D. The green and red fluorescence presented living and dead cells, respectively. The cells distributed on the scaffold. After 24 h, the cells gradually migrated and proliferated into the interior, even proliferated into each layer of the scaffold after seeding 72 h.

At 24 h and 72 h, the proliferation was evaluated and the number of cells was counted by software (Figure 4E). The cell survival rate was calculated using the following equation. The number of green fluorescence/(number of red fluorescence + number of green fluorescence) reflects the cell survival rate. The results indicated that 3D printed GelMA scaffolds are promising cell scaffold with excellent biocompatibility to support cell proliferation.

## Discussion

GelMA was successfully synthesized and then fabricated into scaffold by high resolution polarization modulated stereoscopic projection lithography. The resolution of YC-M3D-10 printer was increased to 4,000 × 2,400 and the printing accuracy was improved to 7 μm, which was nearly five times higher than that of the traditional DLP projection lithography with a resolution of 1,920 × 1,080. The printing accuracy effectively realized the microstructure of gelatin scaffolds and increased the area-volume ratio of the scaffolds. At the same volume, it can provide a larger internal surface area of the scaffold for cell adsorption. Theoretically, the increase of cell density can promote organ function. Compared with the experimental results of G. Eke et al., in 2017, the printed 3D of ADSC cells cultured with the same GelMA material. The average proliferation rate of cells on the scaffold for 1–3 days was 15.7%, while the proliferation ratio of hydrogel was about 4–6% (Eke et al., 2017). The experimental results show that the scaffold has a layered structure to support stem cell growth. The results indicated that cell proliferated significantly on the printed GelMA scaffold after 72 h. Precise GelMA scaffolds printed by YC-M3D-10 can promote cell growth and show great prospects in future tissue engineering applications. In future studies, tissue and cell induction and *in vivo* implantation in mice will be done to further verify the role of organ-like tissue produced by gelatin microscaffolds in implantation *in vivo*.

## Data Availability

The raw data supporting the conclusions of this article will be made available by the authors, without undue reservation.
